# Molecular Analysis of the HOXA2-Dependent Degradation of RCHY1

**DOI:** 10.1371/journal.pone.0141347

**Published:** 2015-10-23

**Authors:** Laure Bridoux, Noémie Deneyer, Isabelle Bergiers, René Rezsohazy

**Affiliations:** From the Animal Molecular and Cellular Biology group (AMCB), Life Sciences Institute (ISV), Université catholique de Louvain, Louvain-la-Neuve, Belgium; Instituto Gulbenkian de Ciência, PORTUGAL

## Abstract

The homeodomain transcription factor Hoxa2 interacts with the RING-finger type E3 ubiquitin ligase RCHY1 and induces its proteasomal degradation. In this work, we dissected this non-transcriptional activity of Hoxa2 at the molecular level. The Hoxa2-mediated decay of RCHY1 involves both the 19S and 20S proteasome complexes. It relies on both the Hoxa2 homeodomain and C-terminal moiety although no single deletion in the Hoxa2 sequence could disrupt the RCHY1 interaction. That the Hoxa2 homeodomain alone could mediate RCHY1 binding is consistent with the shared ability all the Hox proteins we tested to interact with RCHY1. Nonetheless, the ability to induce RCHY1 degradation although critically relying on the homeodomain is not common to all Hox proteins. This identifies the homeodomain as necessary but not sufficient for what appears to be an almost generic Hox protein activity. Finally we provide evidence that the Hoxa2-induced degradation of RCHY1 is evolutionarily conserved among vertebrates. These data therefore support the hypothesis that the molecular and functional interaction between Hox proteins and RCHY1 is an ancestral Hox property.

## Introduction

*Hoxa2* belongs to the extremely well-conserved *Hox* gene family which includes 39 members in mammals. Mammalian *Hox* genes are organized in four clusters located on different chromosomes and can be classified in 13 paralogue groups based on their sequence similarities and their relative position within the clusters. Hox genes code for transcription factors which fulfill well-documented functions during embryonic development. In particular, their most spectacular activities are associated to the patterning of the main body axis, limb development and organogenesis (reviewed in [1]). *Hox* gene activity is also involved in the control of multiple cell behaviors such as proliferation, migration, differentiation or apoptosis (reviewed in [2]). Concordantly with these important roles, *Hox* gene misregulation has been linked to the onset of cell pathologies, namely cancers [3].

Although these genes encode homeodomain transcription factors, a growing body of evidence supports that HOX proteins could also perform non-transcriptional activities. Indeed, their involvement in mRNA translation, DNA repair, initiation of DNA replication and possibly modulation of signal transduction has been highlighted in distinct although still restricted instances (reviewed in [4]).

RCHY1, a protein we recently identified as an interactor of Hoxa2 [5], is a RING-finger type E3 ligase promoting ubiquitination and degradation of different targets. Several of its known substrates, including p53, p27^Kip1^, polH, CHK2 and c-MYC, are known to be involved in the control of cell proliferation and cell death. Consequently, by influencing the abundance of these targets, RCHY1 has been shown to be a regulator of DNA damage response and cell cycle progression (reviewed in [6, 7]). RCHY1 itself is a short-lived protein regulated by the proteasomal degradation pathway and distinct modulators have been reported to negatively influence RCHY1 stability [8, 9]. Indeed, RCHY1 can be self-ubiquitinated leading to its proteasomal processing [10–13]. Moreover, the CaMKII protein kinase was shown to hyper-phosphorylate RCHY1 during the G2/M phases of the cell cycle which, in turn, enhances RCHY1 self-ubiquitination and turnover. Finally, SCYL1BP1 can relocalize RCHY1 to the cytoplasm and promote its ubiquitin-dependent degradation [14].

In a former study, we reported that Hoxa2 expression correlated with a decrease in RCHY1 protein levels. We provided evidence that the RCHY1 decay induced by Hoxa2 involved the proteasome pathway but in an ubiquitin-independent way. Finally, correlatively to the RCHY1 degradation, Hoxa2 expression was also shown to decrease the ubiquitination of p53, in turn, increasing its abundance [5].

Here, we further investigated the process of Hoxa2-induced RCHY1 degradation. We showed that HOXA2 induces a two-fold reduction in RCHY1 half-life, and that this degradation requires the 26S proteasome. Furthermore, we provide evidence that the ability to interact with RCHY1 is shared by HOX proteins although the ability to induce RCHY1 decay is not. We also show that the HOXA2-RCHY1 interaction and RCHY1 degradation are evolutionarily conserved processes, from fish to mammals. Through the analysis of truncated proteins, we reveal that both the homeodomain and the C-terminal extremity of Hoxa2 are indispensable to induce RCHY1 degradation.

## Material and Methods

### Plasmid constructs

Gateway® entry vectors for HOXB1, HOXB2 and p27^Kip1^ and Gateway® expression vectors for VN^173^HOX, VN^173^- and GST-fused Hoxa2 deletion variants have been described elsewhere [15].

Gateway® expression vectors for FLAG-RCHY1 and GST-Hoxa2 [5], the pCMVlacZ [16], pCMV-PBX1a [17], pCS2-Prep1 [18], the Hoxa2 r4-enhancer luciferase reporter [19] and the pKS-Hoxa2 [20] plasmids have been described previously. Gateway® entry vectors (pEnt) for human HOX (except *HOXB1* and *HOXB2*) genes were obtained from the hORFeome v7.1 (http://horfdb.dfci.harvard.edu/hv7/). Mouse *Rchy1* (#MMM1013-202768860), zebrafish *Rchy1* (# MDR1734-202835821), xenopus *Rchy1* (#MXL1736-202784859) and xenopus *Hoxa2* (MXL1736-202785685) templates were purchased from GE Healthcare. Chicken *Rchy1* was amplified from cDNA obtained from a chick embryo at Hamburger-Hamilton Stage 30. Obtained sequence was different from the reference chicken *Rchy1* (NM_001080888.1) sequence displaying two silent mutations, C108T and C264T. The sequence was submitted to GenBank under the accession number KT375311.

Sequences coding for Hoxa2 and Rchy1 from distinct vertebrate species were PCR-amplified using primers described in Table 1. The resulting PCR products were inserted into the pDON223 vector using the Gateway® Technology from Invitrogen to generate the corresponding pEnt vectors (Table 1).

**Table 1:** Primers used to generate pEnt plasmids.

Plasmid	Forward primer (5’-3’)	Reverse primer (5’-3’)
pEntRchy1gallus	GGGGACAACTTTGTACAAAAAAGTTGGCATGGCGGCTGTCGGGGa	GGGGACAACTTTGTACAAGAAAGTTGGGTACTCCACAGGCTGCTTGG
pEntRchy1mus	GGGGACAACTTTGTACAAAAAAGTTGGCATGGCGGCGACGGCGc	GGGGACAACTTTGTACAAGAAAGTTGGGCACTGCTGATCCACTGGC
pEntRchy1xenopus	GGGGACAACTTTGTACAAAAAAGTTGGCATGGAGGTGACGGGAGGc	GGGGACAACTTTGTACAAGAAAGTTGGGCATTCCCTGGCTGTCTGC
pEntRchy1zebrafish	GGGGACAACTTTGTACAAAAAAGTTGGCATGGCCGCCACGAAAGTG	GGGGACAACTTTGTACAAGAAAGTTGGGCAGTGGTGCTGCTCGGCA
pEntHoxa2gallus	GGGGACAACTTTGTACAAAAAAGTTGGCATGAATTTCGAATTCGAGCGA	GGGGACAACTTTGTACAAGAAAGTTGGGCAGTAATTCAGATGCTGTAAG
pEntHoxa2xenopus	GGGGACAACTTTGTACAAAAAAGTTGGCATGAATTACGAATTTGAGCGAGA	GGGGACAACTTTGTACAAGAAAGTTGGGCAGTAGTTCAAATGGTGCAAG
pEntHoxa2zebrafish	GGGGACAACTTTGTACAAAAAAGTTGGCATGAATTACGAATTCGAGCGA	GGGGACAACTTTGTACAAGAAAGTTGGGTAGTAGCTCAAGTGTTGCAG

The resulting pEnt plasmids were confirmed by DNA sequencing and used to generate pExp mammalian expression vectors for FLAG-tagged or GST-tagged proteins with the v1899 destination vector and pDest-GST N-terminal destination vector [21], respectively. pExp vectors for fusion proteins involving the VN^173^ and VC^155^ Venus protein moieties were obtained with pDest-VN^173^ [22] and pDest-VC^155^ [22], respectively.

The DNA sequence corresponding to the *Rchy1 in situ* hybridization (ISH) probe was PCR-amplified from genomic DNA using the following primers, CAGCGTGAACACCTGAGGAT and GCTTATGTTGTCACAAGAGCCA and cloned into the pCR2.1 TOPO plasmid using the TOPO® TA Cloning® Technology (Invitrogen).

### Cell culture, transfection and treatments

Cultured cells were maintained at 37°C, in a humidified atmosphere with 5% CO_2_. HEK293T cell line was grown in Dulbecco’s Modified Eagle Medium (D-MEM) with Gultamax-I (#61965, GIBCO) supplemented with 10% fetal bovine serum (#10270–106, Invitrogen), 100 U/ml of penicillin-streptomycin (#15140–122, GIBCO) and 1 mM sodium pyruvate (11360–070, GIBCO). COS-7 cells were maintained in Dulbecco’s Modified Eagle Medium (D-MEM) (#31885–023, GIBCO) supplemented with 10% fetal bovine serum and 100 U/ml of penicillin-streptomycin. Twenty-four hours after cell plating, plasmid constructs were transfected with jetPRIME transfection reagent (#114–07, Polyplus-transfection) according to the manufacturer's instructions. For proteasome inhibition, 24 h after transfection, cells were treated with 5–10 μM MG132 dissolved in DMSO (#474790, Calbiochem), 1 μM of b-AP15 dissolved in DMSO or with DMSO alone as negative control, for periods of 6–15 h. For half-life measurements, 24 h after transfection, the proteasome was inhibited for 4 h as previously described, then treated with 200 μg/ml of cycloheximide (#01810, Sigma) dissolved in DMSO following different exposure times.

### Protein abundance analysis and western blotting

To evaluate the impact of HOX expression on RCHY1 abundance, HEK293T were transfected with distinct combinations of expression vectors, at 500 ng each. Cells were lysed for 20 minutes at 4°C in ice-cold IPLS lysis buffer (0.5% NP-40, 20 mM Tris-HCl pH 7.5, 0.5 mM EDTA, 120 mM NaCl, 10% glycerol) containing protease inhibitor cocktail (#11873580001, Roche). Cells lysates were centrifuged for 5 minutes at 1000 g at 4°C. Supernatants were recovered and equal amounts of proteins were boiled 5 minutes at 95°C in Laemmli loading buffer (10% SDS, 30% glycerol, 350 mM Tris-Cl pH 6.8, 600 mM DTT, 0.1% bromophenol blue) before loading SDS-PAGE for electrophoresis. Proteins were then transferred onto a nitrocellulose membrane (#10600002, Amersham Biosciences). Membranes were blocked in 10% low-fat milk. Anti-FLAG (M2) (#F1804, Sigma) and anti-GST primary antibodies (#G1160, Sigma) were used at a 1: 5000 dilution in 1% milk. Goat anti-mouse secondary antibody was HRP-conjugated and used at a 1: 10 000 dilution (#sc-2005, Santa Cruz). For β-ACTIN detection, HRP-conjugated anti-β-ACTIN was used at a 1: 20 000 dilution (#A3854, Sigma). Finally, membranes were treated with a chemiluminescence detection system (#NEL104001EA, PerkinElmer) and exposed to a photographic film. Relative protein quantification was carried out using ImageJ software.

### Bimolecular Fluorescence Complementation assay (BiFC)

COS-7 cells were cultured on glass coverslips and transfected with distinct combinations of pExp-VN^173^ and pExp-VC^155^ vectors for the fusion proteins and/or pDest-VN^173^ and pDest-VC^155^ empty controls, each at 500 ng. When indicated, the proteasome was inhibited as previously described. Twenty-four to 31 h after transfection, cells were rinsed in PBS solution and fixed for 20 minutes with 4% paraformaldehyde (PFA) in PBS, then rinsed twice in TBS (50 mM Tris, 155 mM NaCl, pH 7.5) containing 0.1% Triton X100. Coverslips were then used for immunofluorescence detection of proteins or rinsed once in TB (50 mM Tris pH 7.5) prior to mounting.

### Immunofluorescence

Cells transfected for BiFC assay were cultured on glass coverslips, rinsed in PBS and fixed for 20 minutes with 4% PFA in PBS. Cells were further blocked with 10% low-fat milk or 10% BSA in TBS-0.1% Triton X100 solution for 30–45 minutes at RT depending on the antibody (milk, anti-PML and anti-SC35; BSA, anti-γH2AX), before overnight incubation in TBS-0.1% Triton X100-1% low-fat milk or BSA solution at 4°C, with anti-PML (ab53773, Abcam), anti-SC35 (556363, Becton Dickinson) or anti-γH2Ax (#9718P, Cell Signaling Technologies) antibody, respectively, used at 1: 80. Cells were rinsed three times for 10 minutes in TBS-0.1% Triton X100 and incubated with Alexa Fluor® 488 Donkey Anti-Rabbit (A-21206, Life technologies) in TBS-0.1% Triton X100 solution for 45 minutes at RT. Finally, cells were rinsed twice with TBS-0.1% Triton X100 and once with TB (50 mM Tris pH 7.5).

### Imaging

Glass coverslips were mounted in Vectashield®-DAPI medium (Vector laboratories). Slides were then analyzed by epifluorescence (Axioskop 2, Zeiss) or confocal microscopy (LSM710, Zeiss, Jena, Germany). Fluorescence signals were quantified using ImageJ software. BiFC fluorescence from the test and the control conditions were quantified and the interaction was considered positive when the tested interaction emitted at least 3 times more fluorescence than the 3 control conditions.

### β-Galactosidase and Luciferase assays.

HEK293T cells were transfected with 250 ng of reporter plasmid, and 50 ng of pCS2-PREP1, pCMV-PBX1a and/or expression vector for full length GST-Hoxa2 or deletion GST-Hoxa2 derivatives. To avoid experimental variations due to transfection efficiency, an internal standard reporter corresponding to the *lacZ* gene under the control of a constitutive CMV promoter (pCMVlacZ, [21]) was also added in cotransfection experiments (25 ng). Cells were harvested 48 h after transfection for enzymatic assays. Lysis and enzymatic activity dosages were performed with the β-Gal reporter gene assay kit (#11758241001, Roche) and the Luciferase reporter gene assay kit (#11 669893001, Roche), respectively. Luciferase activity was then normalized to that of β-Galactosidase.

### ISH, RNA extraction and RT-PCR from mouse embryos

All animal experiments were performed in accordance with the guidelines established by the Animal Experimentation Ethics Committee of the Université catholique de Louvain and in agreement with the European directive 2010/63/UE (approval number 103002). Mice were maintained and fed under standard conditions on a 14 h light / 10 h dark cycle. All the experiments were carried out on adult CD1 female mice mated overnight with adult CD1 males. When plugs were detected, pregnant mice were killed between 8.5 to 12.5 days post coitum by gas inhalation and embryos were rapidly dissected while kept on ice. Embryos for ISH were rinsed in PBS, fixed overnight with 4% PFA in PBS at 4°C, rinsed three times 20 minutes in PBS before cryopreservation by incubation at 4°C, for 2 h in 10% sucrose/PBS and then overnight in 20% sucrose/PBS. Thereafter, embryos were embedded in OCT (Shandon CryomatrixTM, Thermo Electron, France), frozen on dry ice and stored at -80°C. Using a Leica CM 3050S cryostat, seven sets of 20 μm thick serial transversal or sagittal cryosections per embryo were obtained. Gene expression was detected using digoxigenin-labeled RNA probes as previously described by Hutlet et al. [23]. For probe synthesis, the pKS-Hoxa2 and the pCR2.1-TOPO-Rchy1 plasmids were linearized with EcoRI and SpeI, and the probes were transcribed with the T3 and the T7 polymerases, respectively. Hybridized sections were analyzed on a Leica DM2500 microscope and pictures were captured with a Leica DFC420C camera.

Embryos for RT-PCR were frozen in liquid nitrogen and stored at -80°C. Total RNA was extracted with the High Pure RNA Isolation Kit (Roche) according to the manufacturer's instructions. RNA was reverse transcribed using a reaction mix containing 200 ng random hexamer primers (#SO142, Life technologies), 1 mM dNTP (#R0191, Life technologies), 10 U riboLock RNase inhibitor (#EO0381, Life technologies), 100 U RevertAid Reverse transcriptase and the provided buffer (#EP0441, Life technologies). The mixture was incubated for 10 minutes at 25°C, 1 h at 42°C and 10 minutes at 80°C. Specific intron-spanning primers listed in Table 2 were designed based on NCBI database sequences. For *Rchy1* and *Actin* amplifications, PCR reaction mix contained 1.25 U Taq DNA Polymerase (#EP0402, Life technologies) with the provided buffer supplemented with 1.9 mM MgCl_2_, 250 μM dNTP (#R0191, Life technologies) and 1.25 mM of each primer. The amplification program started with an activation step at 95°C for 5 minutes followed by 35 cycles of denaturation at 95°C for 30 seconds, hybridization at 57°C for 15 seconds and elongation at 72°C for 45 seconds. The last cycle was completed by a final elongation step at 72°C for 7 minutes. For *Hoxa2* amplification, PCR reaction mix contained 1 U Expand Long Template (Roche) with the provided buffer (n°1), 250 μM dNTP (#R0191, Life technologies) and 250 nM of each primer. The amplification program started with an activation step at 95°C for 5 minutes followed by 35 cycles of denaturation at 95°C for 30 seconds, hybridization at 55°C for 15 seconds and elongation at 68°C for 45 seconds. The last cycle was completed by a final elongation step at 68°C for 7 minutes.

**Table 2:** Primers used for RT-PCR reactions.

TARGETED GENES	Forward primer (5’-3’)	Reverse primer(5’-3’)
*Actin*	CCACCATGTACCCAGGCATT	AGGGTGTAAAACGCAGCTCA
*Hoxa2*	AGACCTCGACGCTTTCACAC	TGGTTTTCCTTGCACTGGGT
*Rchy1*	TGAGGTAGCACAGACTCCCA	ACTCTGCGTGCATAGTATCACTT

### Statistical analysis

All statistical analyses were performed with JMP11 software. Mixed model, followed by a post hoc a Dunnett’s test, was used for statistical purposes. Western blot quantifications were analyzed using the gel as a random parameter, the tested HOX or the tested deletion Hoxa2 derivatives as fix parameters, Ln(RCHY1/ACTIN) intensity as the response and the GST condition as the control. Luciferase activation was analyzed using the experiment as a random parameter, the tested deletion Hoxa2 derivatives as a fix parameter, Log(Luciferase/βGal) as the response and “PREP-PBX” or “Hoxa2-PREP-PBX”conditions as the controls.

## Results

### RCHY1 half-life is decreased by HOXA2 in a 26S proteasome-dependent way

**Fig 1:**
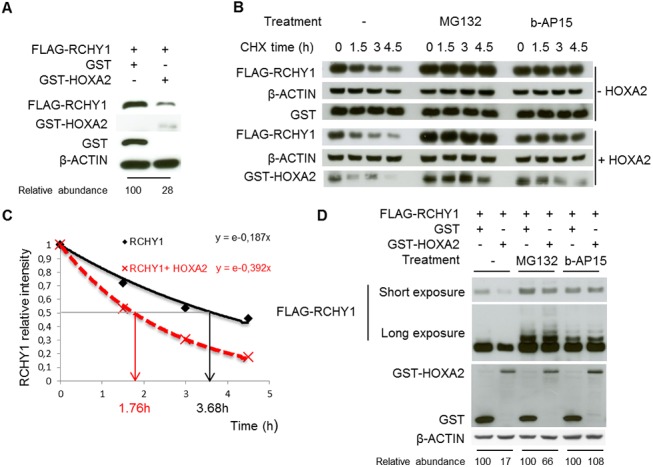
HOXA2 reduces RCHY1 half-life in a 26S proteasome-dependent way. (A-B-D) HEK293T cells were cotransfected with expression vectors coding for FLAG-RCHY1 and GST-HOXA2 or GST. Cell lysates were then subjected to immunoblot analysis with antibodies against FLAG, GST and β-ACTIN. The relative RCHY1 abundance was determined with regards to that of β-ACTIN. (B) To determine the RCHY1 half-life in the absence (-HOXA2) or in presence (+HOXA2) of HOXA2, cells were treated with cycloheximide (CHX) for the indicated time. The involvement of the proteasome in the RCHY1 degradation was assayed by treating cells with proteasome inhibitors (MG132 or b-AP15) for 4 hours prior to inhibition of protein translation with CHX. (C) The intensity of the RCHY1 and β-ACTIN bands observed upon cycloheximide treatment, without proteasome inhibition, was quantified and the relative RCHY1/β-ACTIN ratios were plotted. (D) The involvement of the proteasome in the HOXA2-induced RCHY1 degradation was assayed by treating cells with proteasome inhibitors (MG132 or b-AP15) for 6 hours. Upon prolonged exposition time, western blot with anti-RCHY1 recognizes higher molecular weight bands probably associated to ubiquitinated forms of RCHY1.

Further to an interactomic screen carried out by Bergiers et al. to identify Hoxa2 partner proteins, the Hoxa2 protein from the mouse was shown to interact with the human RCHY1 and to induce its destabilization [5]. To confirm these data obtained with proteins of heterologous origin, we addressed whether this destabilization process was conserved for the human proteins. To this end, we transiently transfected HEK293T cells to express human FLAG-RCHY1 and GST or human GST-HOXA2 fusion proteins. As shown in Fig 1A, the level of RCHY1 was considerably decreased in the presence of HOXA2 supporting that, like for the murine Hoxa2, expression of the human HOXA2 had a negative effect on RCHY1 protein accumulation.

To further investigate the effect HOXA2 exerts on RCHY1, we first quantified RCHY1 half-life and then assayed the impact of Hoxa2 on this parameter. HEK293T cells were transiently transfected with a FLAG-RCHY1 expression vector. Twenty four hours later, *de novo* protein synthesis was inhibited using cycloheximide (CHX) (200 μg/ml) and cells were collected at different time-points (0, 1.5, 3 and 4.5 h). Western blot analysis revealed that RCHY1 protein levels decreased within 3 h and more dramatically after 4.5 h (Fig 1B). Densitometry analysis indicated that RCHY1 half-life (with quantification normalized to β-ACTIN levels) was about 3.68 h, which is consistent with previously reported data [8, 9] (Fig 1C). Thereafter, to address to what extent HOXA2 protein might impact on RCHY1 half-life, GST-HOXA2 was transfected in combination with FLAG-RCHY1. Upon co-expression, RCHY1 protein levels were drastically reduced. Indeed, HOXA2 caused a significant 2-fold reduction in RCHY1 half-life, which was decreased to 1.76 h (Fig 1B and 1C).

Similarly to previous reports by Bergiers et al. [5], we addressed whether HOXA2-dependent reduction in RCHY1 stability was the result of proteasome-mediated degradation. We thus compared RCHY1 levels and half-life in the presence or absence of proteasome inhibition. Upon transfection of FLAG-RCHY1 and subsequent treatment with the 20S proteasome inhibitor MG132 (5–10 μM) for 6 h, RCHY1 protein levels were dramatically increased, whereas β-ACTIN levels remained unaltered. Moreover, the destabilization of RCHY1 induced by HOXA2 was significantly abolished by MG132 treatment therefore confirming that HOXA2 induces RCHY1 degradation through a proteasome-dependent pathway (Fig 1B and 1D). In addition, multiple higher molecular weight bands revealed by the anti-FLAG antibody appeared upon MG132 treatment which likely correspond to ubiquitination of RCHY1 (Fig 1D). Such post-translational modifications of RCHY1 have indeed been assayed and confirmed previously [5].

Most of the substrates degraded by the 26S proteasome are polyubiquitinated. However, in a limited number of cases, degradation of non-ubiquitinated proteins by the 20S core proteasome has been reported [24–26]. For example, 14-3-3τ, MDM2, NQO1 and Rchy1 respectively promote p21, RB, p53 and PolH turnover through the 20S proteasome independently of the ubiquitination status of their substrates [24, 27, 28]. In this model, the destabilization of p21 and RB was shown to be promoted by their interaction with PSMA3, an α-subunit of the 20S core proteasome [27, 29, 30]. This hypothesis suggests that for their degradation, non-ubiquitinated proteins could bypass the 19S regulator moiety of the proteasome to be directly targeted to the 20S core proteases [29]. We previously reported that Hoxa2 could destabilize RCHY1 independently of its ubiquitination and that Hoxa2 was capable of interacting with the PSMA3 and PSMB2 subunits of the 20S core particle [5]; supporting a similar mechanism to the Hoxa2-mediated decay of RCHY1 as described above. To investigate whether RCHY1 bypasses the 19S cap proteasome and is directly targeted to the 20S core proteasome by HOXA2, the activity of the 19S was inhibited with b-AP15. This drug inhibits the deubiquitinating activity of both ubiquitin C-terminal hydrolase 5 (UCHL5) and ubiquitin-specific peptidase 14 (USP14), two constitutive proteins of the 19S proteasome, leading to an accumulation of polyubiquitinated proteins [31]. High molecular weight forms of RCHY1 likely corresponding to ubiquitinated proteins were detected upon b-AP15 treatment (1 μM) supporting the drug’s efficiency. However, our results showed that the RCHY1 reduction induced by HOXA2 could be rescued by b-AP15 (Fig 1D). Moreover, exposure to b-AP15 also resulted in an enhanced stability of RCHY1 after cycloheximide treatment (Fig 1B). These data suggest that 19S cap proteasomal activity is required for the HOXA2-mediated RCHY1 decay.

In conclusion, RCHY1 half-life has been estimated to be around 3.5 h and is dramatically reduced upon expression of HOXA2. The RCHY1 turnover is mediated by the proteasomal pathway and the function of both the 20S core and 19S cap proteasome is required.

### HOXA2 and RCHY1 mainly interact in the nucleus

**Fig 2:**
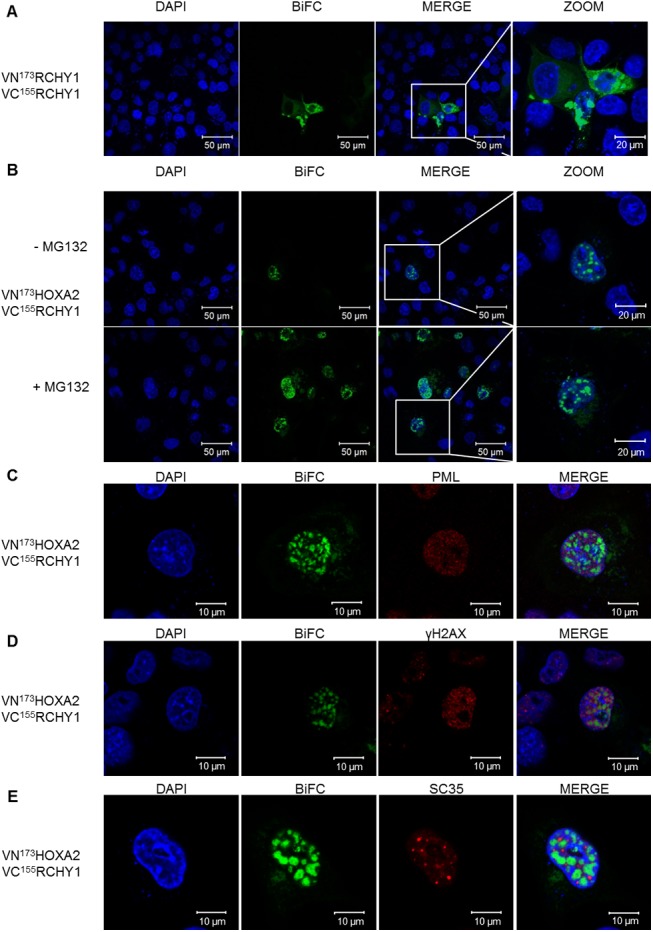
BiFC assay reveals RCHY1 and HOXA2 mainly interact in the nucleus. COS-7 cells were transfected with VN^173^HOXA2 or VN^173^RCHY1 coding vectors together with VC^155^RCHY1 coding vector. Upon interaction between the partner proteins, the VN^173^ and VC^155^ moieties of the Venus fluorescent protein brought together provide a fluorescent signal. (A) Cells were transfected with vectors coding for VN^173^RCHY1 and VC^155^RCHY1. (B) Cells were transfected with VN^173^HOXA2 and VC^155^RCHY1 then treated or not with MG132 proteasome inhibitor. (C-E) Cells were transfected with VN^173^HOXA2 and VC^155^RCHY1 for BiFC, treated with MG132 proteasome inhibitor and subjected to immunofluorescence with anti-PML (C) anti-γH2AX (D), or SC35 (E). Nuclei were stained with DAPI (blue).

The localization of the RCHY1-HOXA2 interaction was studied using BiFC assay which not only validates the possible direct interaction between two partner proteins but can also indicate where it takes place in live cells or *in vivo*. BiFC relies on the ability of N- and C-terminal parts of the Venus protein to emit detectable fluorescence once they are brought in close proximity. The HOXA2 protein was fused downstream of the N-terminal 173 amino acids of Venus (VN^173^), while RCHY1 was C-terminally fused to the C-terminal moiety of Venus (amino acids 155 to 243; VC^155^). Controls supporting that the N- and C-terminal Venus fragments did not reassociate if not fused to interacting proteins were also involved. Consistently, the VN^173^HOXA2/VC^155^, the VN^173^/VC^155^RCHY1 and the VN^173^/VC^155^ combinations showed little to no fluorescence (S1 Fig). As preliminary controls, BiFC was first assayed for two well-established RCHY1-mediated interactions: RCHY1 dimerization and the RCHY1-p27^Kip1^ interaction [12, 32, 33]. The VN^173^RCHY1 and VC^155^RCHY1 fusion proteins provided diffuse and punctate fluorescence mainly in the cytoplasm as well as a weaker diffuse signal in the nucleus (Fig 2A). In addition, the majority of the cells transfected with VN^173^RCHY1 and VC^155^p27^Kip1^ provided a diffuse signal both in the cytoplasm and the nucleus concordantly with known regulation of p27^Kip1^ by RCHY1 in these subcellular compartments (S2 Fig) [33]. It is of note that a few cells presented a particular punctate staining in the cytoplasm for the VN^173^RCHY1 and VC^155^p27^Kip1^ interaction (S2 Fig).

The vast majority of the emitted BiFC signal associated to the HOXA2-RCHY1 interaction localized in the nucleus and most frequently appeared to be punctate. Moreover, the number of fluorescent cells and signal intensity were drastically increased by treating cells with proteasome inhibitor MG132, confirming the involvement of the proteasomal pathway (Fig 2B). The background fluorescence produced by the control conditions was weak compared to the corresponding test condition, as observed in S1 Fig. To identify the nuclear sub-compartment stained by the punctate signal associated to the RCHY1-HOXA2 interaction, we tried to colocalize the BiFC signal with distinct immunolabeled nuclear domains.

First, PML bodies were targeted. These structures are dynamic nuclear foci 0.2–1 μm wide consisting in multimolecular platforms where recruited proteins together with post-translational modifiers act to modulate protein activation, sequestration or degradation. Cellular processes such as transcription, response to DNA-damage or resistance to micro-organisms, for example, have been shown to be regulated within these nuclear structures (reviewed in [34]). Preliminary data suggested that Hoxa2 could be relocalized to the PML bodies upon inhibition of the proteasome (I. Bergiers, unpublished data). However, our results show that the BiFC staining did not overlap with PML immunoreactivity (Fig 2C).

Second, DNA repair foci were analyzed since RCHY1 has been shown to play a role in DNA repair. Notably, RCHY1 is involved in the degradation of PolH, a DNA polymerase required for DNA repair [28, 35]. Moreover, IR-induced cell death was found to be altered in RCHY1 knockout mice [36]. For this reason, it was of interest to look at γH2AX, a marker of DNA repair foci. As shown in the Fig 2D, the BiFC signal emitted by the RCHY1-HOXA2 interaction did not coincide with the pattern observed for γH2AX.

Finally, we targeted nuclear speckles with an antibody against the splicing factor SC35. These structures contain RNA splicing machinery also including transcription factors and notably HOXA1 [37]. No overlaps were observed in the SC35 staining pattern and the HOXA2-RCHY1 interaction.

In conclusion, the HOXA2-RCHY1 interaction appears to mainly take place in the nucleus and is enhanced upon proteasomal inhibition. Though we excluded PML bodies, DNA repair foci and nuclear speckles, the actual nuclear subcompartment where the interaction localizes remains to be identified.

### The HOXA2-induced destabilization of RCHY1 is an evolutionarily conserved phenomenon

**Fig 3:**
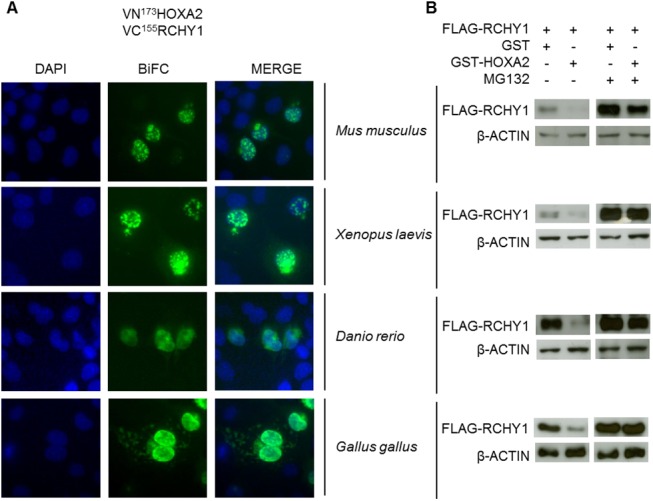
The RCHY1-HOXA2 interaction and HOXA2-induced degradation of RCHY1 are evolutionarily conserved processes. (A) COS-7 cells were transfected with different combinations of vectors coding for VN^173^HOXA2 and VC^155^RCHY1 originating from different vertebrate species and treated with the MG132 proteasome inhibitor for 6 h. Nuclei were stained with DAPI (blue). (B) HEK293T cells were cotransfected with different combinations of expression vectors coding for FLAG-RCHY1 and GST or GST-HOXA2 from different vertebrate species and treated or not with the MG132 proteasome inhibitor. Cell lysates were then subjected to immunoblot analysis with antibodies recognizing FLAG and β-ACTIN.

Since the HOX family of proteins is extensively conserved among bilaterian animals, we next questioned whether RCHY1 destabilization induced by HOXA2 is evolutionarily conserved among vertebrates. RCHY1 and HOXA2 coding sequences from mouse, chicken, xenopus and zebrafish were cloned and each HOXA2 orthologue was tested for its ability to interact with and destabilize the RCHY1 protein from autologous origin. Like the human proteins, mouse, chicken, xenopus and zebrafish HOXA2 orthologues shared the ability to interact with RCHY1 (Fig 3A) and promote its degradation (Fig 3B). As previously reported, RCHY1 destabilization induced by HOXA2 results from proteasome-mediated degradation [5]. We therefore addressed whether the destabilization was differentially influenced upon proteasome inhibition by MG132 for the HOXA2/RCHY1 orthologues. For all HOXA2-RCHY1 protein pairs, RCHY1 decay was indeed blocked by proteasomal inhibition (Fig 3B). Hence, we conclude that the proteasome-dependent destabilization of RCHY1 by HOXA2 is an evolutionarily conserved phenomenon.

### Rchy1 is expressed during mouse embryogenesis

*Hoxa2* expression and functions are well known to take place from the gastrulation stage on during embryogenesis [38, 39]. Beitel (2002) and Leng (2003) [11, 40] have previously reported that *Rchy1* is differentially expressed in various adult mouse tissues. However, data concerning *Rchy1* expression during mouse development are currently lacking. For this reason, it was of interest to investigate whether *Rchy1* expression pattern overlaps with that of *Hoxa2* in the embryo.

**Fig 4:**
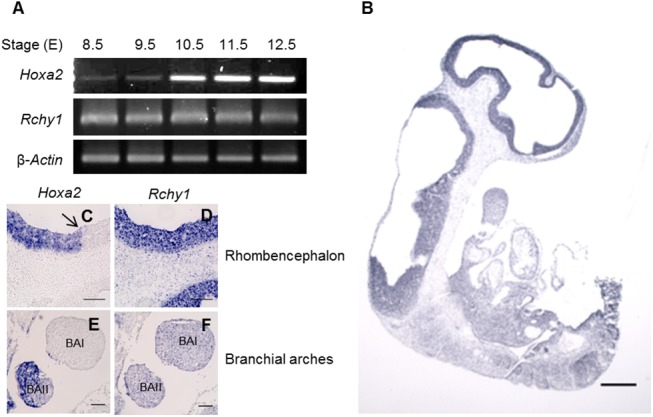
*Rchy1* and *Hoxa2* expression patterns overlap during mouse embryogenesis. (A) Detection of *Hoxa2*, *Rchy1* and *β-Actin* transcripts by RT-PCR in mouse embryos from E8.5 to E12.5. ISH of *Rchy1* (B-D-F) and *Hoxa2* (C-E) on sagittal cryosections of mouse embryos at E10.5. The black arrow indicates the boundary between the r1 and r2 rhombomeres. BAI; first branchial arch, BAII, second branchial arch. Scale bar = 100 μm.

We first analyzed *Rchy1* expression in the developing embryo from E8.5 to E12.5, stages at which *Hoxa2* expression is well described. RT-PCR on different cDNA pools confirmed *Rchy1* expression at all the tested embryonic stages (Fig 4A). ISH on sagittal sections at E10.5 revealed a widespread but heterogeneous staining. In particular, strong signal was detected in the anterior neuroepithelium while it appeared weaker posteriorly (Fig 4B). We then compared *Hoxa2* and *Rchy1* expression patterns focusing on the hindbrain, at the boundary between rhombomeres (r)1 and 2, and the branchial arches, which are structures affected in *Hoxa2* knockout mice. While *Hoxa2* expression showed a clear limit at the r1-r2 junction, staining for *Rchy1* overlapped with the *Hoxa2* expression domain and extended more rostrally, towards the midbrain (Fig 4C and 4D). Similarly, while *Hoxa2* was specifically expressed in the second but not in the first branchial arches, *Rchy1* expression was detected in both of them (Fig 4E and 4F).

In conclusion, our results show that *Rchy1* is expressed during mouse embryogenesis and highlight that both *Hoxa2* and *Rchy1*, previously identified as genes coding for interacting proteins in cultured cells, are expressed in overlapping territories at the same embryonic stages.

### Mapping the Hoxa2 domains involved in interacting and destabilizing RCHY1

**Fig 5:**
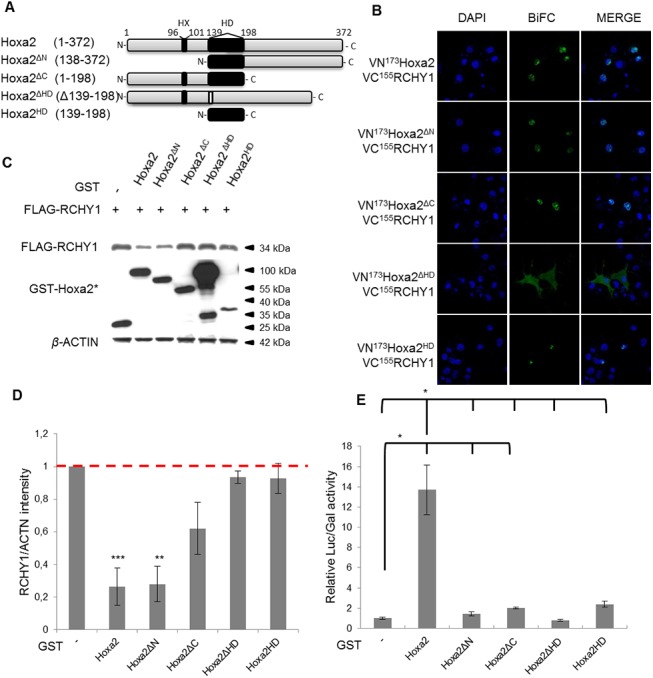
Involvement of the homeodomain and C-terminal half of Hoxa2 in promoting RCHY1 degradation. (A) Schematic representation of Hoxa2 deletion constructs lacking the N-terminal domain, the C-terminal domain or the homeodomain (HD) (HX: hexapeptide). (B) COS-7 cells were transfected with different combinations of vectors coding for VC^155^RCHY1 and VN^173^Hoxa2 or VN^173^Hoxa2 deletion derivatives, and treated with the MG132 proteasome inhibitor. Nuclei were stained with DAPI (blue). (C) HEK293T cells were cotransfected with expression vectors coding for FLAG-RCHY1 and GST, GST-HOXA2 or GST fused Hoxa2 deletion derivatives. Cell lysates were then subjected to immunoblot analysis with antibodies targeting FLAG, GST or β-ACTIN. (D) The RCHY1 and β-ACTIN proteins detected as in (C) were quantified and the relative RCHY1/β-ACTIN abundance was compared to the control condition involving the unfused GST protein for which the RCHY1/β-ACTIN ratio was set at 1 (dashed red line). Bars indicate the standard deviation (3 < n < 4). (E) HEK293T cells were transfected with a Luciferase (Luc) reporter construct containing a Hoxa2-responsive element and a constitutive β-Galactosidase (Gal) reporter as standard. Expression vectors for PBX1a, PREP1 and GST-tagged Hoxa2 variants were added in combination. The Luc/Gal activity ratios were compared relatively to that obtained with the control transfection involving the unfused GST protein (relative activity of 1). Bars indicate the standard deviation (N = 2, n = 6). (D-E) Asterisks indicate a significant difference between ratios for unfused GST and GST-HOX conditions (Dunnett, * = 0.01 < P < 0.05, ** = 0.001 < P < 0.01, *** = 0.0001 < P < 0.001).

In order to determine which regions of the Hoxa2 protein are involved in RCHY1 interaction and destabilization, we designed four Hoxa2 deletion variants in which residues from the N-terminal (Hoxa2^ΔN^ [138–372]), C-terminal (Hoxa2^ΔC^ [1–198]), both terminal domains (Hoxa2^HD^ [139–198]), or the homeodomain (Hoxa2^ΔHD^ [δ139–198]) were removed (Fig 5A). We then assayed these variants for their interaction with RCHY1 by BiFC. Hoxa2 and the deletion mutants were fused to VN^173^, while RCHY1 was fused to VC^155^. Emitted fluorescence was observed with all the tested mutants suggesting that an extended or at least two separate Hoxa2 protein regions are capable of establishing contacts which are sufficient but not absolutely necessary to the RCHY1-Hoxa2 interaction, i.e. subsets of contacts are sufficient to support the interaction (Fig 5B). Nonetheless, our results indicate that some contacts at least could be mapped to the homeodomain and others to the C- and/or N-terminal moieties of Hoxa2.

Next, the GST-fused Hoxa2 variants were individually cotransfected with FLAG-RCHY1 and the effect of each deletion on the HOXA2-mediated destabilization of RCHY1 was tested. As shown in Fig 5C and 5D, the N-terminal deletant still promoted RCHY1 destabilization. In contrast, both the C-terminal and the homeodomain deletants failed to significantly affect RCHY1 stability indicating that these regions are required to promote RCHY1 degradation (Fig 5C and 5D). Consistently, the homeodomain alone did not induce RCHY1 decay. We therefore conclude that the Hoxa2 fragment spanning aa138-372 contains the necessary elements for RCHY1 destabilization. However, as Hoxa2^ΔC^, Hoxa2^HD^ and Hoxa2^ΔHD^ were all able to interact without leading to RCHY1 destabilization, it can also be concluded that the interaction between the two partners is not sufficient to lead to RCHY1 degradation.

To further determine whether Hoxa2’s impact on RCHY1 abundance is associated to its transcriptional activity, we tested if Hoxa2 deletion variants were still active in transcription. Luciferase assays were carried out with a reporter construct under the control of a Hoxa2-responsive element we previously characterized as an auto-regulatory enhancer active in the developing hindbrain [19]. Expression vectors for the Hoxa2 cofactors Pbx1a and Prep1 were added in the assay to provide full activation of the reporter. All the tested Hoxa2 deletions highly or completely compromised Hoxa2 transcriptional activity with regards to the reporter gene (Fig 5E). This therefore provides evidence that the N-terminal, HD and C-terminal domains of the protein are necessary for an efficient transcriptional activity. As a corollary, since the Hoxa2 variant missing the N-terminal part was almost inactive in transcription (Fig 5E), but remained functional for RCHY1 destabilization (Fig 5C and 5D), we conclude that the Hoxa2-mediated degradation of RCHY1 is independent of its transcription factor activity and can be considered as a new non-transcriptional function for a Hox protein.

### RCHY1 is bound by multiple HOX proteins but destabilized by only few of them

**Fig 6:**
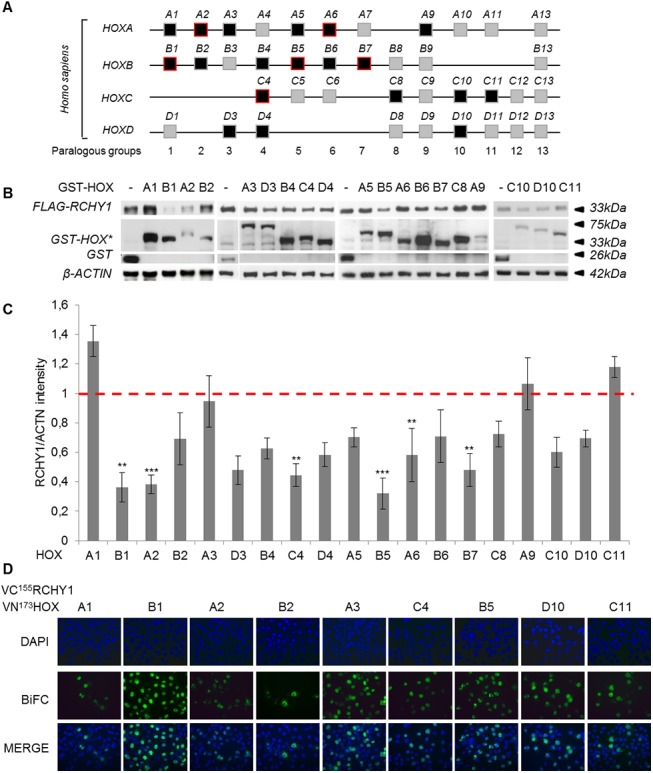
Several human HOX proteins induce RCHY1 degradation. (A) Schematic representation of the 4 human HOX complexes and 13 groups of paralogue genes. Black boxes indicate the HOX proteins assayed and red frames the proteins showing a significant negative effect on RCHY1 stability. (B) HEK293T cells were cotransfected with expression vectors coding for GST or GST-tagged HOX and FLAG-RCHY1. Cell lysates were then subjected to immunoblot analysis using FLAG, GST or β-ACTIN antibodies. (C) The RCHY1 and β-ACTIN proteins detected as in (B) were quantified and the relative RCHY1/β-ACTIN abundance was compared to the control condition involving the unfused GST protein for which the RCHY1/β-ACTIN ratio was set at 1 (dashed red line). Bars indicate the standard deviation (3 < n < 12). Asterisks indicate a significant difference between ratios for unfused GST and GST-HOX conditions (Dunnett, * = 0.01 < P < 0.05, ** = 0.001 < P < 0.01, *** = 0.0001 < P < 0.001). (D) COS-7 cells were transfected with different combinations of vectors coding for VN^173^HOX and VC^155^RCHY1 for BiFC analysis and treated with the MG132 proteasome inhibitor. Nuclei were stained with DAPI (blue).

Our Hoxa2-deletion analysis revealed that the homeodomain is involved both in the HOXA2-RCHY1 interaction and in the induction of RCHY1 degradation. Since HOX proteins share important sequence conservation, in particular in their homeodomain, we addressed whether HOX proteins other than HOXA2 were capable of interacting and destabilizing RCHY1. Nineteen out of the 39 *HOX* genes were subcloned to code for GST fusion proteins and they were individually cotransfected with FLAG-RCHY1 in HEK293T cells (Fig 6A and 6B). Cell lysates were collected and processed to estimate relative RCHY1 stability (Fig 6B). Western blot and densitometry analyses revealed that RCHY1 abundance varied upon GST-HOX expression and that RCHY1 was significantly destabilized by HOXB1, HOXC4, HOXB5, HOXA6 and HOXB7 (Fig 6C). These results indicate that several HOX proteins, but not all of them, can induce RCHY1 decay similarly to HOXA2. HOXB1, HOXC4 and HOXB5 interaction with RCHY1 was further confirmed by BiFC assay upon MG132 treatment and the corresponding emitted fluorescent signal localized in the nucleus (Fig 6D).

We then addressed whether the inability of some HOX proteins to destabilize RCHY1 was due to lack of interaction. Surprisingly, while assaying HOXA1, HOXB2, HOXA3, HOXC11 and HOXD10, which did not show a significant impact on RCHY1 stability, these proteins appeared to interact with RCHY1 upon MG132 treatment (Fig 6D).

In conclusion, in light of these results, we hypothesize that binding to RCHY1 is a general property shared by HOX proteins but that the ability to promote RCHY1 degradation is restricted to a subset among them.

## Discussion

While HOX proteins are known to be involved in a vast range of activities, their molecular interactions have been poorly characterized to date. In a previous study, we identified RCHY1, an E3 ubiquitin-ligase, as a new interactor of Hoxa2 and provided data supporting that Hoxa2 promotes the proteasomal degradation of RCHY1. Here, we reported (1) that the HOXA2-RCHY1 interaction mainly takes place in the nucleus (2) that the RCHY1 decay induced by HOXA2 depends on both the 19S and the 20S proteasome particles, (3) that the interaction involves molecular determinants from at least two distinct HOXA2 regions providing contacts which are sufficient but not necessary for the binding of RCHY1 and (4) that some HOXA2 deletion derivatives, though still capable of interacting with RCHY1, have lost the ability to provoke its protesomal degradation. Finally, the results provided in the present study support that the downregulation of RCHY1 provoked by HOXA2 is conserved among orthologues from other vertebrate species and that binding to RCHY1 seems to be generic characteristic of HOX proteins yet the ability to stimulate its protesomal degradation is shared only by a subset of them.

The N-terminal part of Hoxa2 seems dispensable to the induction of RCHY1 degradation. Indeed, the Hoxa2 deletion mutant lacking the N-terminal portion of the protein destabilizes RCHY1 to the same extent as the WT Hoxa2. This result could be related to what we previously showed for the Hoxa2^WMAA^ protein mutated in the hexapeptide sequence located in the N-terminal part of Hoxa2. Similarly to Hoxa2^ΔN^, the Hoxa2^WMAA^ protein has been shown to induce RCHY1 decay [5]. This hexapeptide motive is known to mediate the interaction of Hox proteins with PBX [41] and in the case of Hoxa2, we provided evidence that amino acid substitutions in the hexapeptide severely impaired or abolished the transcriptional activity of Hoxa2 [19]. As both Hoxa2^ΔN^ and Hoxa2^WMAA^ mutants lack the hexapeptide sequence but maintain full activity with regards to RCHY1 destabilization, we propose that the Hoxa2-mediated effect on RCHY1 degradation is independent of the transcription activity of Hoxa2 and corresponds to a novel non-transcriptional activity for Hoxa2.

Conversely, although all the HOXA2 variants tested so far remain capable of interacting with RCHY1, the ability to induce RCHY1 destabilization is only affected upon deletion of the C-terminal part or the entire homeodomain of HOXA2, or as a consequence of point mutations in the homeodomain (Hoxa2^KQN-RAA^), as we previously reported [5]. This suggests that the molecular determinants involved in inducing RCHY1 degradation are distinct from the ones sustaining the interaction. This also demonstrates that integrity of the homeodomain is necessary for RCHY1 degradation. However, although necessary, the homeodomain is not sufficient to act on RCHY1 turnover.

In accordance with the observation that the homeodomain is sufficient to mediate the interaction with RCHY1, all other tested HOX proteins were shown to be able to interact with RCHY1. Moreover, consistently with the fact that the homeodomain alone is insufficient to promote RCHY1 decay, the majority of the tested HOX proteins did not display any significant impact on RCHY1 decay, with only 6 out of the 19 HOX proteins tested leading to a significant destabilization of RCHY1. This again supports that the interaction between HOX and RCHY1 proteins does not necessarily lead to RCHY1 degradation and that the molecular determinants involved in inducing RCHY1 degradation are distinct from those sustaining the interaction. We could therefore hypothesize that an additional domain might be required for RCHY1 destabilization. However, protein alignments of HOXB1, A2, C4, B5, A6 and B7 did not enable us to identify common motives that are absent from HOX proteins showing no effect on RCHY1 stability and that could be linked to this function.

Similar molecular interactions generic to HOX proteins have already been described, including TALE-class homeodomain transcription factors Pbx and Meis, CUL4 ubiquitine-ligase, histone acetyltransferase CPB or pre-replication complex inhibitor Geminin [41–49]. At a functional level, some generic activities have also been highlighted such as the inhibition of autophagy in drosophila [50]. Molecular properties and activities often appear largely shared among proteins belonging to the same paralogue group. For example, Hox proteins of paralogue group 3 have been shown to be functionally interchangeable [51]. However, although the interaction with RCHY1 appears common to several if not all HOX proteins, its functional consequence in terms of modulating RCHY1 stability does not seem to be neither generic to HOX protein nor specific to one HOX since only a subset of HOX proteins tested could induce a significant RCHY1 destabilization. Remarkably, this activity is not paralog-specific but is instead shared by proteins corresponding to genes spread along the vertebrate HOX complexes. Determining why a given HOX protein can impact on RCHY1 stability or not deserve further investigation.

In addition to the RCHY1 degradation mediated by several HOX proteins we report here, two other studies previously reported HOX involvement in promoting protein degradation. Namely, HOXB4 and HOXA9 associate with Roc1-Ddb1-Cul4a ubiquitin ligase and contribute to Geminin ubiquitination leading to its proteasomal degradation [47, 52]. Several similarities can be highlighted between the degradation processes described in these studies and the present report. Similarly to what we observed for HOXA2, the integrity of the HOXB4 HD was shown to be required to promote Geminin decay. Furthermore, as for RCHY1, the ability to interact with Geminin is shared by multiple HOX proteins, conversely to the ability to destabilize it. Indeed, Geminin was shown to be destabilized by HOXB4 and HOXA9 but not by HOXC13 [5, 47, 52].

Since HOXA2-induced RCHY1 degradation is evolutionarily conserved in all the tested vertebrates and this activity is shared by several HOX from different paralogue groups, we hypothesize that controlling RCHY1 stability is an ancestral HOX function. Whether ancestral HOX proteins displayed the ability to induce RCHY1 destabilization is an issue that could be addressed by testing if this activity is shared among HOX proteins from Bilaterian clades other than vertebrates.

The proteasomal degradation of RCHY1 promoted by HOXA2 was shown to occur independently of the ubiquitin system[5]. Ubiquitin-independent protesomal degradation of proteins has already been reported in several instances [24–30]. The direct involvement of the 20S core particle has been implicated in this process and was confirmed for RCHY1 using MG132, a drug that blocks the proteolytic activity of this part of the proteasome. It was proposed that Hoxa2 interacts with the 20S core proteasome to directly induce RCHY1 degradation. However, whether the 19S cap proteasome was required for such ubiquitin-independent protein decay remained unknown. Using b-AP15, a drug specifically inhibiting the 19S cap proteasome [31], we observed an abolishment of the HOXA2-induced RCHY1 degradation supporting that the 19S also is implicated in this degradation pathway.

Finally, to our knowledge, this is the first report and description of *Rchy1* expression during mouse embryogenesis. *Rchy1* expression shows an extended pattern with a stronger staining detected in the anterior neuroepithelium. Most significantly, the overlap between *Hoxa2* and *Rchy1* gene expression supports the possibility of an interaction between these proteins during mouse embryogenesis. Though *Rchy1* expression appears to be dispensable for normal embryonic development as suggested by the phenotype of the knockout mouse [36], the protein has been shown to differentially regulate its targets under stressed and unstressed conditions and might play a more predominant role under stressed conditions during embryonic development as well.

## Supporting Information

S1 Fig BiFC control conditions. COS-7 cells were transfected with vectors coding for VN^173^HOXA2 and VC^155^RCHY1; VN^173^ and VC^155^RCHY1; VN^173^HOXA2 and VC^155^; VN^173^ and VC^155^. Only the VN^173^HOXA2 and VC^155^RCHY1 combination provides a BiFC signal. Nuclei were stained with DAPI (blue).
(TIF)

S2 Fig BiFC assay of the RCHY1-p27^Kip1^ interaction. COS-7 cells were transfected with the VN^173^RCHY1 coding vector together with the VC^155^p27^Kip1^ coding vector. Nuclei were stained with DAPI (blue).
(TIF)
