# Supplementary figures and images for: Molecular Analysis of the HOXA2-Dependent Degradation of RCHY1
Source: PLoS One. 2015 Oct 23;10(10):e0141347. doi: 10.1371/journal.pone.0141347 (PMC4619689; doi:10.1371/journal.pone.0141347)

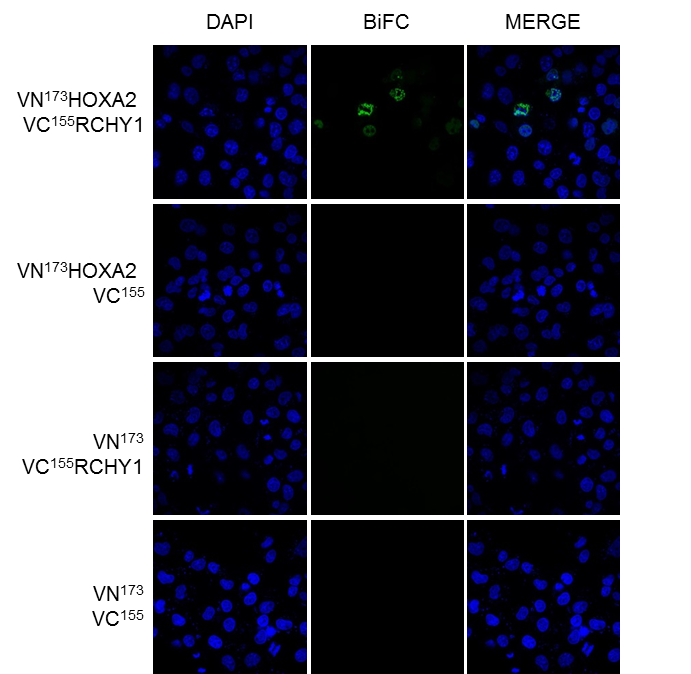

Supplement: S1 Fig — COS-7 cells were transfected with vectors coding for VN173HOXA2 and VC155RCHY1; VN173 and VC155RCHY1; VN173HOXA2 and VC155; VN173 and VC155. Only the VN173HOXA2 and VC155RCHY1 combination provides a BiFC signal. Nuclei were stained with DAPI (blue). (TIF) [file pone.0141347.s001.tif]

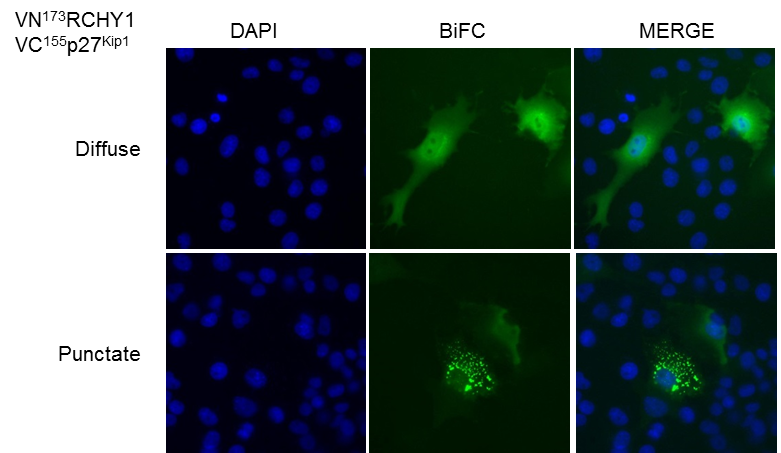

Supplement: S2 Fig — COS-7 cells were transfected with the VN173RCHY1 coding vector together with the VC155p27Kip1 coding vector. Nuclei were stained with DAPI (blue). (TIF) [file pone.0141347.s002.tif]
